# Errors in Names of 2 Individuals in Text

**DOI:** 10.1001/jamanetworkopen.2026.32867

**Published:** 2026-08-12

In the Invited Commentary titled “Regulating the Level of Nicotine in Combustible Cigarettes,”^1^ published July 15, 2026, the name of the then-Commissioner of the US Food and Drug Administration is David Kessler, not Richard Kessler. The surname of the corresponding author of the Original Investigation that is the subject of the commentary is Denlinger-Apte, not Derlinger-Apte. This article has been corrected.^1^

## References

[1] Vander Weg MW. Regulating the level of nicotine in combustible cigarettes. JAMA Netw Open. 2026;9(7):e2622551. doi:10.1001/jamanetworkopen.2026.22551 42455576

